# Beyond the gift: unveiling the impact of donor-recipient relationship on donors’ quality of life and mental health – results of a european bi-center study

**DOI:** 10.3389/ti.2026.16536

**Published:** 2026-09-02

**Authors:** Christina Papachristou, Natalia Papoulaki, Entela Kondi, Klemens Budde, Xavier Torres, Nina Babel, Ignacio Revuelta

**Affiliations:** 1 Department of Psychology, Aristotle University of Thessaloniki, Thessaloniki, Greece; 2 Department for Psychosomatic Medicine, Charité University Medicine, Berlin, Germany; 3 DTI Foundation, Barcelona, Spain; 4 Clinic for Nephrology CCM, Charité University Medicine, Berlin, Germany; 5 Hospital Clinic de Barcelona, Barcelona, Spain; 6 Department for Internal Medicine Campus Virchow, Charité-University Medicine, Berlin, Germany; 7 Department of Nephrology and Kidney Transplant, Hospital Clinic Barcelona, Barcelona, Spain

**Keywords:** caregiver role, donor-recipient relationship, living kidney donation, long-term psychosocial follow-up, psychosocial outcome

## Abstract

While living kidney donation is considered safe and beneficial, long-term psychosocial donor outcomes remain under-explored, particularly regarding donor-recipient relationships and caregiving dimensions. This study examined quality of life (QoL) and psychological outcomes among 713 living kidney donors from two major European transplant centers, assessing QoL, anxiety, depression and somatization alongside donor-recipient relationship types, caregiver role, caregiving burden and perceived responsibility for the recipient’s health. After adjusting for gender, age, center and time since donation, relationship type was not associated with physical wellbeing, but was linked to lower mental wellbeing in spouse donors compared to child-to parent and other genetically related donors. Caregiver role was linked to lower mental wellbeing and increased anxiety, caregiving burden with higher somatization, and perceived responsibility for recipient’s health with higher depression. Identified differences in mental health between centers likely reflect cultural and organisational characteristics, while differences in wellbeing linked to older age and time since donation are eventually understood within ageing processes. Conclusively, caregiving burden and perceived responsibility for the recipient’s health emerged as key predictors of psychological distress, while the effect of relationship type on wellbeing remains inconclusive. While most donors demonstrate good psychosocial outcomes, at-risk subgroups warrant appropriate attention prior and after donation.

## Graphical Abstract

**Figure:**
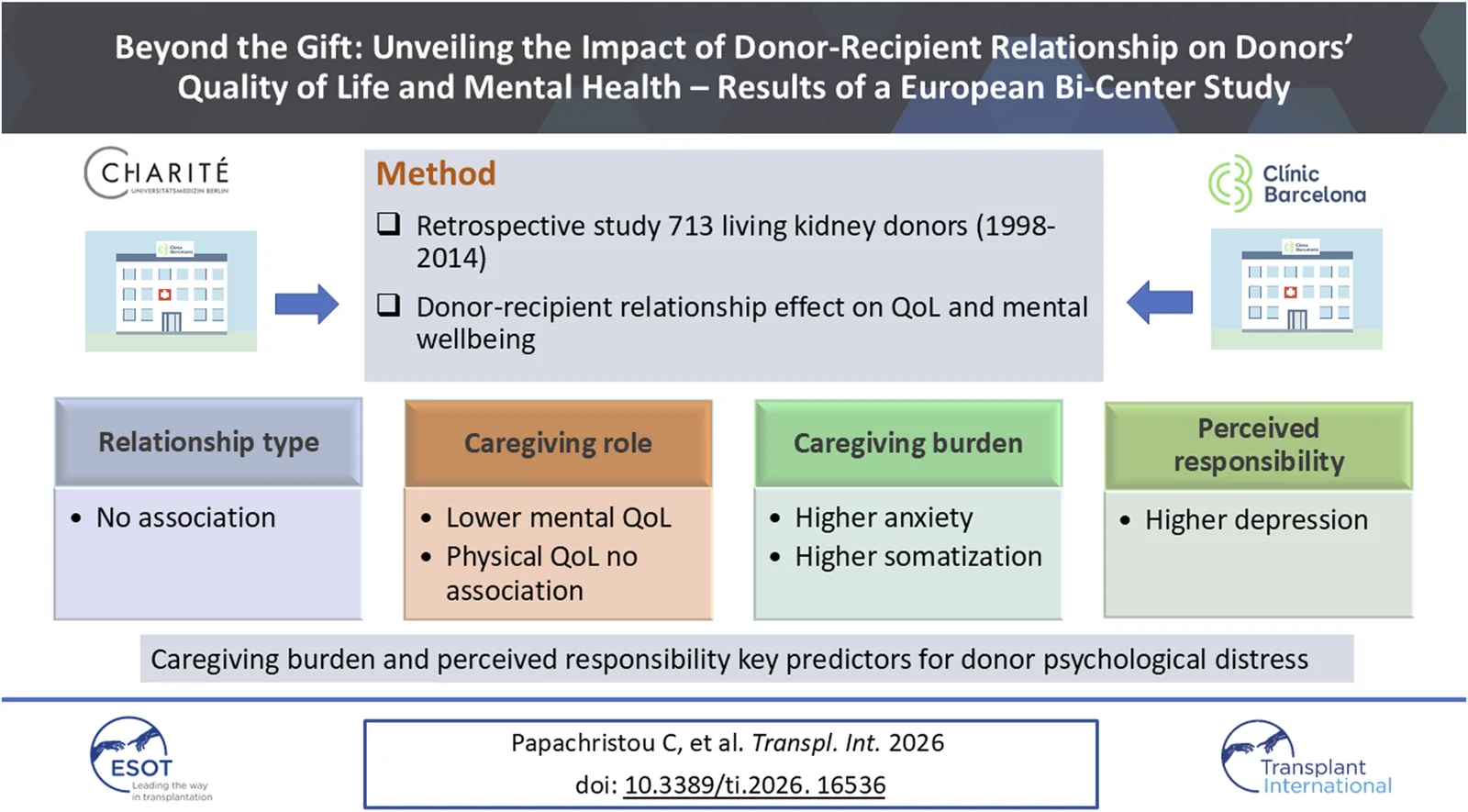

## Introduction

Living kidney donor transplantation (LKDT) is the optimal treatment for patients with end-stage renal disease providing better quality of life and cost-effectiveness compared to deceased kidney donor transplantation and chronic dialysis treatment [1–5]. Living kidney donors are mostly motivated by the desire to assist the recipient [6] and any inducement to increase LKDT rates should be practiced with the expectation that donors’ risk is outweighed by the recipient’s health benefits. Data show that donors prioritize the relationship with the recipient as the fifth most significant aspect of donation, subsequent to factors such as kidney function, personal recovery, complications, and family life [7]. Both quantitative and qualitative studies indicate that the donor-recipient bond either remains stable or strengthens after donation, reinforcing the initial motivation to donate [6–10]. However, a closer relationship post-donation is not always the case. Approximately 2%–6% of donors may report a deterioration in their relationship with the recipient [10–16]. Issues arising in the donor-recipient relationship may turn convalescence into a distressing condition including unresolved tensions [17], a conflicting dual role of being both donor and caregiver for the recipient, concerns regarding the recipient’s health or that the recipient endangers the donated organ, unmet expectations surrounding recovery, graft loss and recipient’s death [6, 7, 18–23].

Although the donor-recipient bond is related to the donors’ physical and mental health post-donation [8, 24–31], only a few studies delve into the donor-recipient relationships [6, 8, 9, 24, 31]. The donor-recipient relationship type - be it a parent-to-child or a child-to-parent donation, or a donation among siblings or spouses - appears to shape the psychological experience of donors, as different roles, dependencies or expectations describe different relationship types. And even though the care giving role burden seems to ameliorate after transplantation [32] non-met expectations, especially in spousal dyads, lead to disappointment affecting donors satisfaction [8, 33]. Additionally, much of the existing literature is based on single-center studies with small sample sizes [24, 34, 35] and predominantly focuses on short-term follow-up periods, typically spanning from several months to one-year post-donation [18, 24, 26, 28, 31, 34]. Long-term data about donor-recipient relationship types are limited [25, 30]. The growing heterogeneity of donors’ profiles including non-related, unspecified and altruistic donors highlights the need for further research into the role of the donor-recipient type on the donor wellbeing and psychosocial outcome [36–38]. Furthermore, while QoL, anxiety and depression have been the most used measures to assess the mental health of living donors, somatization has been largely overlooked as an eventually important measure. Somatization as a process is based on the notion that distress when not able to be processed through emotional and verbal processes is expressed somatically. Pistorio et al. [39] in their study with recipients point out to this mechanism suggesting that emotional distress related to the transplant relationship is handled through neurotic or immature defenses and may be more likely to express itself somatically. While this could apply also to donors - who through their “protective” role towards the recipients might be additionally inhibited in their emotional and verbal expression of distress - this aspect has not been studied so far. While the SoLKiD study has been designed with this awareness there is yet no substantial body of findings published that map the donor-recipient relationship on the somatized burden [40].

In this paper, we are exploring the association between different donor-recipient relationship types and the associated burden of care giving as well as the feeling of responsibility for the recipient with the physical and mental quality of life of donors, anxiety, depression and somatization rates. The data used for the analysis were collected within the European Living Donor Psychosocial Follow-Up (ELIPSY) project in two major kidney transplant centers in Europe regarding the long-term quality of life and psychosocial outcome of living kidney donors. The project was funded by the Executive Agency for Health and Consumers under grant agreement 20081104 and received ethical approval from the relevant Scientific Ethical Committees for Clinical Research.

## Patients and methods

### Study design, participants and data collection

**FIGURE 1:**
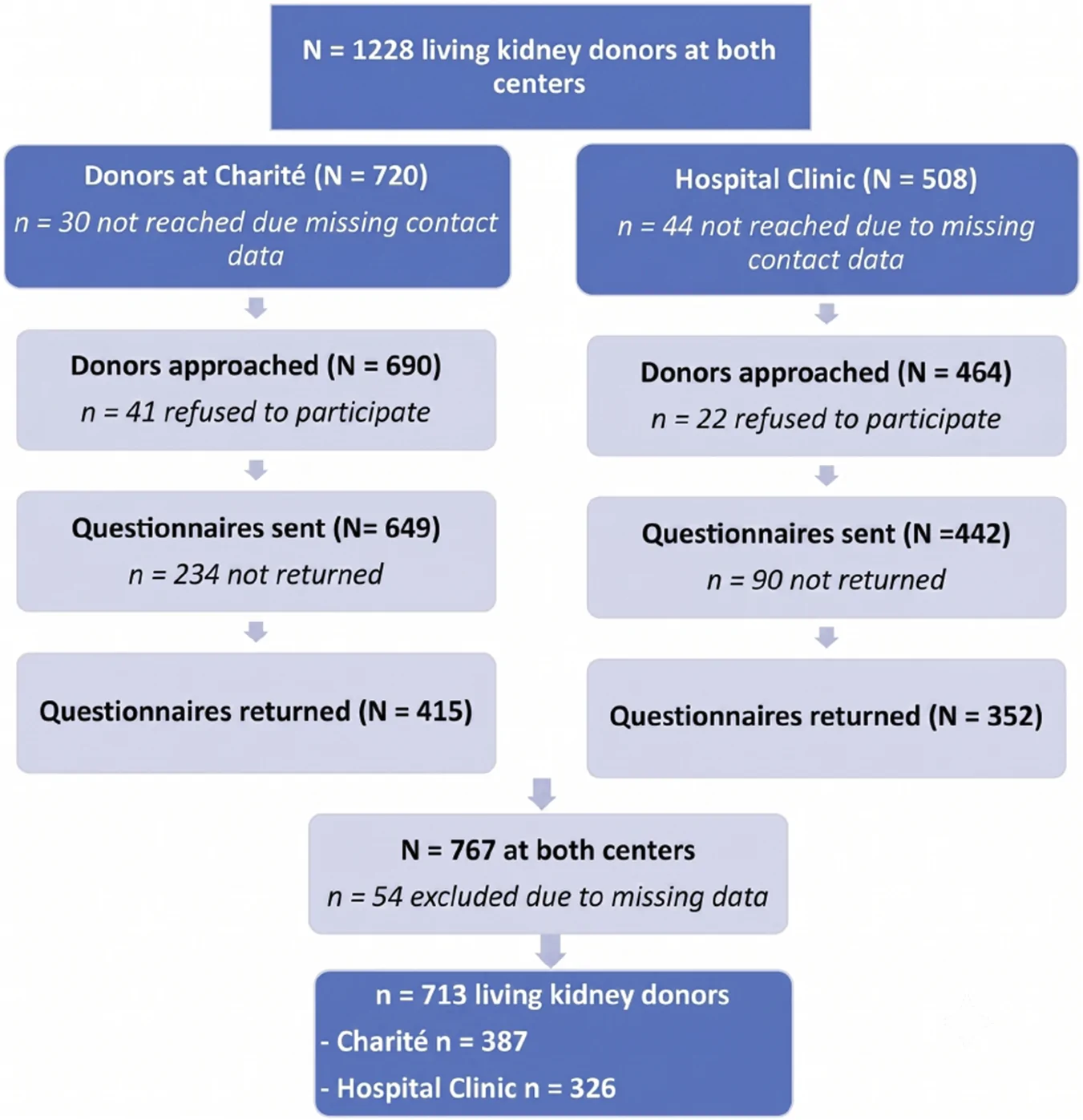
Donors’ enrollment flow chart.

This retrospective study is part of the ELIPSY project, which aimed to evaluate the psychosocial outcomes and quality of life of a long cohort of living kidney donors across six European transplant centers (Spain, Germany, France, Portugal, Sweden, and Turkey). Due to insufficient sample sizes at some sites, the present analysis included data only from the kidney transplant units of the Hospital Clinic of Barcelona (Spain) and Charité University Medicine Berlin (Germany). At both centers, donors and recipients underwent comprehensive pre-donation medical and psychological assessments. Participants were eligible if aged 18 years or older and had adequate language proficiency of the respective country to complete the questionnaires. All individuals who donated a kidney at either center between 1998 and 2014 were contacted initially by telephone. Upon consent and home address confirmation, participants received a mailed study package including information, consent forms, a self-administered questionnaire and a prepaid envelope to return the signed consent form and completed questionnaire. If the questionnaire was not returned within two to 3 weeks, donors received a telephone follow-up reminder. Data collection took place over 2 years. A total of 1,228 potential participants were identified through hospital records. 1154 were contacted as 74 had missing contact details or were living abroad. 63 actively refused participation on the phone. Questionnaires were sent to 1,091 participants and 767 returned the questionnaire (response rate 66.46%). Subsequently, 54 participants (7%) were excluded from analysis because of missing responses resulting in a final sample of 713 participants. No significant differences were observed between included and excluded participants regarding sex, center, time since donation or anxiety rate (PHQ). However, those excluded reported 1.44 lower mean depression rate (PHQ) than those included [t (704) = −2.10, (−2.78, −0.10) p = 0.036] and those included were older than those excluded [t (765) = 2.38, (0.61, 6.39) p = 0.017] with a mean difference 3.50 (1.47). An overview of the enrollment process is shown in Figure 1.

Donors’ psychosocial outcome assessed via the above mentioned self-administered structured questionnaire, incorporated both standardized psychometric instruments and donation-specific items. Standardized tools were selected based on established validity and frequent use in international research, allowing cross-study comparison. The ad-hoc items were developed based on clinical experience and previous research, including the European Living Donation and Public Health (EULID) project [41].

### Standardized instruments

Donors’ quality of life was assessed using the 36-Item Short-Form Health Survey (SF-36) [42]. This instrument comprises of eight subscales: physical functioning (PF; Cronbach’s *α* = 0.90), general health (GH; Cronbach’s *α* = 0.74), bodily pain (BP; Cronbach’s *α* = 0.86), role limitations due to physical health problems (RP; Cronbach’s *α* = 0.87), social functioning (SF; Cronbach’s *α* = 0.83), vitality (VT; Cronbach’s *α* = 0.85), role limitations due to mental health problems (RE; Cronbach’s *α* = 0.89), and emotional wellbeing (MH; Cronbach’s *α* = 0.84). A physical component summary (PCS) score was calculated by averaging the first four subscales (Cronbach’s *α* = 0.93), and a mental component summary (MCS) score was calculated by averaging the latter four subscales (Cronbach’s *α* = 0.92). For each subscale, item scores were coded, averaged, and transformed into a 0–100 scale, where higher scores indicated better health. A score of 50 serves as the reference value [43].

The Patient Health Questionnaire (PHQ) [44] was employed to assess depression, anxiety, and somatization. The depression module (PHQ-D; *α* = 0.85) includes nine items, reflecting the nine diagnostic criteria for depression from the Diagnostic and Statistical Manual of Mental Disorders, Fourth Edition [45]. Each item is rated from 0 (not at all) to 3 (nearly every day), producing a total score between 0 and 27. The anxiety module (PHQ-A; *α* = 0.93) consists of seven items [46], rated on a 3-point Likert scale (0 = not at all, 2 = more than half the days), yielding a total score ranging from 0 to 21. The somatization module (PHQ-S; *α* = 0.79) includes 13 items [47], rated from 0 (not bothered at all) to 2 (bothered a lot), with a total score ranging from 0 to 26. Scores of 5, 10, and 15 marking the cutoffs for mild, moderate, and severe depression, anxiety and somatization respectively.

### Ad-hoc items

Ad-hoc items assessed whether donors assumed a caregiving role for the recipient (“Have you been in charge of the recipient since the donation?” yes/no) and the subjective caregiving burden (“If yes, how much burden was for you taking care of the recipient?” 10-point scale). Additional questions evaluated donors’ subjective feelings of responsibility for the recipient’s illness or death (“Somehow I feel responsible for the recipient’s death or bad condition, please leave unchecked, if that does not apply to the recipient” yes/no).

### Statistical analysis

All statistical analyses were performed using IBM SPSS Statistics, Version 27.0 (SPSS Inc., Chicago, IL, USA). Categorical variables are presented as frequencies (*n*) and percentages (%), whereas continuous variables are expressed as means (*M*) and standard deviations (*SD*). Clinical variables were compared across groups using one-way analysis of variance (ANOVA) with *post hoc* comparisons, as well as Student's *t*-test, as appropriate. General linear models were used to examine the associations between donor–recipient relationship type and physical and mental wellbeing. In addition, multiple linear regression analyses were performed to investigate the associations of the caregiving role or responsibility for the recipient’s health with the endpoints of interest (QoL, anxiety, depression, somatization), adjusting for relevant covariates. To account for multiple testing, p-values from the primary multivariable analyses were adjusted using the Benjamini–Hochberg false discovery rate (FDR) procedure. The remaining analyses are reported in the Supplementary Material/Appendix as exploratory findings and are presented without FDR adjustment. To ensure transparency and minimize the risk of selective reporting, both significant and non-significant exploratory results are provided. Statistical significance was set at p < 0.05.

## Results

Data from a total sample of 713 living kidney donors were analyzed; 326 participants (45.7%) donated at the Hospital Clinic Barcelona and 387 (54.3%) at the Charité University Medicine Clinic of Berlin. Table 1 illustrates the sociodemographic characteristics of the donors, separated by center.

**TABLE 1:** Sociodemographic characteristics of living kidney donors separated by centre (N = 713).

Characteristics	All donors (*n* = 713)	Charité (*n* = 387)	Hospital clinic (*n* = 326)
Age (years), mean (*SD)*	52.73 (10.41)	52,66 (10.12)	52.81 (10.77)
Time since donation (months), median (*SD*), (range)	38 (45.52) (Range = 6–202)	39 (52.38) (Range = 6–202)	36 (33.16) (Range = 10–191)
Gender, *n* (%)			
Male	249 (34.9)	130 (33.6)	119 (36.5)
Female	464 (65.1)	257 (66.4)	207 (63.5)
Marital status, *n* (%)			
Single	69 (9.8)	37 (9.7)	32 (9.9)
Married	498 (70.8)	263 (69.2)	235 (72.8)
Separated	77 (10.9)	44 (11.6)	33 (10.2)
Widowed	57 (8.1)	36 (9.5)	21 (6.5)
Educational status, *n* (%)			
No school degree	12 (1.7)	4 (1)	8 (2.5)
Elementary	322 (45.4)	186 (48.4)	136 (41.7)
High school	207 (29.2)	98 (25.5)	109 (33.4)
University	169 (23.8)	96 (25)	73 (22.4)
Employment status, *n* (%)			
Full-time	280 (39.3)	157 (40.6)	123 (37.7)
Part-time	82 (11.5)	55 (14.2)	27 (8,0.3)
Unemployed/Students	53 (7.4)	25 (6.5)	28 (8.6)
Retired	193 (27.1)	112 (28.9)	81 (24.8)
Homemakers	62 (8.7)	17 (4.4)	45 (13.8)
Type of the relationship with the recipient, *n* (%)			
Parent-to-child	234 (32.8)	142 (36.7)	92 (28.2)
Spouse/Partner	276 (38.7)	163 (42.1)	113 (34.7)
Sibling	136 (19.1)	54 (14)	82 (25.2)
Friend	20 (2.8)	14 (3.6)	6 (1.8)
Child-to-parent/Other genetically related	38 (5.3)	13 (3.4)	25 (7.7)
Unrelated	9 (1.3)	1 (0.26)	8 (2.45)
Allocation, *n* (%)			
Directed and related	702 (98.5)	386 (99.7)	316 (96.9)
Directed and altruistic	1 (0.1)	0	1 (0.3)
Paired and non-directed	7 (1)	1 (0.3)	6 (1.8)
Pooled and non-directed	1 (0.1)	0	1 (0.3)
Nationality, *n* (%)			
German	384 (53.9)	384 (99.2)	-
Spanish	319 (44.7)	-	319 (97.9)
Other (Andorra, Colombia, Ecuador, Turkey, United Kingdom, Vietnam)	8 (1)	3 (0.8)	5 (1.5)

### The donor-recipient relationship

Table 2 demonstrates the link between donor-recipient relationship type on donors’ physical and mental quality of life (PCS, MCS). Although an exploratory ANOVA analysis found a significant relationship between PCS and relationship type, the association was no longer statistically significant after adjusting for confounding variables such as sex, age, months since donation and center. After adjustment, those donated at Hospital Clinic reported higher PCS than those donating at Charité [β = 4.63 (1.37), I (1.93–7.34), p < 0.001]. Older age was related to lower PCS (β = −0.18 (0.07), [−0.31, −0.04], p = 0.010) and longer time since donation [β = −0.06 (0.015), 95% CI [−0.09, −0.03], p < 0.001] was also significantly linked to lower PCS.

**TABLE 2:** Unadjusted and adjusted analyses examining the associations between donor-recipient relationship type, caregiving role, responsibility about recipient’s health and post-donation physical or mental health outcomes (N = 713).

Outcome (Y)	Predictor (X)	Exploratory Test	Exploratory Result	Unadjusted Regression *β* (SE), (95% CI), *R* ^*2*^, *p*	Adjusted* Regression F (df), *β* (SE), (95% CI), R^2^, p	FDR-adjusted p-value
PCS	Type of relationship	ANOVA	*F* (5,613) = 2.97, *p* = .012	*-*	*F* (5,618) = 0.97, *p* = .435, η_p_^2^ = .008	.435
MCS	Type of relationship	ANOVA	*F* (5,700) = 2.37, *p =* .04	-	*F* (5,705) = 2.005, *p* = .076, η_p_^2^ = .014	.106
PCS	Caregiving role	*t*-test	*t* = 1.19, *p* > .05	*β =* -1.91 (1.61), (−5.08,1.26), *R* ^2^ = .002, *p* = .24	*β =* -2.29 (1.57), (−5.37,0.79), *R* ^2^ = .086, *p* = .146	.170
MSC	Caregiving role	*t*-test	*t* (658) *=* 3.19, *p* = .001	*β =* -5.04 (1.58), (−8.14, -1.94), *R* ^2^ = .015, *p* = .001	*β =* -6.06 (1.55), (−9.05, -2.96), *R* ^*2*^ = .084, *p* < .001	<.007
PHQ-A	Burden of caregiving	-	-	*β =* 0.24 (0.09), (0.06,0.42), *R* ^2^ = .052, *p* = .011	*β =* .26 (0.09), (0.08, 0.45), *R* ^*2*^ = .146 , *p* = .005	.018
Somatization	Burden of caregiving	-	-	*β =* 0.24 (0.11), (0.02, 0.46), *R* ^*2*^ *=* .035, *p* = .034	*β* = .28 (0.11), (0.07,0.50), *R* ^*2*^ = .166, *p* = .011	.026
PHQ-D	Responsibility about recipient’s health	*t*-test	*t* (295) *=* 3.10, *p* = .002	*β =* 2.68 (0.86)*,* (0.98,4.37), *R* ^2^ = .03, *p* = .002	*β* = 2.06 (0.86), (0.36,3.75), *R* ^2 =^ .09, *p =* .017	.030

Adjusted for the following confounders: Age, sex, months since donation, center.

A general linear model was conducted to examine the association between type of relationship with the recipient and MCS scores, adjusting for sex, age, months since donation, and transplant center. No significant overall association was observed between relationship type and MCS scores across groups (all adjusted p-values >0.05). Exploratory pairwise comparisons of adjusted means suggested a difference between spouses (M = 75.59, SE = 1.07) and children/other genetically related donors (M = 83.27, SE = 2.89); however, this finding should be interpreted with caution given the absence of a significant overall effect. Controlling the covariates, female donors had lower MCS scores compared with male donors [β = −2.79 (1.41), (−5.56, −0.03), p = 0.048]. Older age at donation was associated with higher MCS scores [β = 0.23 (0.07), (0.10–0.36), p < 0.001], whereas a longer time since donation was associated with lower MCS scores [β = −0.04 (0.015), (−0.07, −0.01), p = 0.014]. In addition, donors recruited from Hospital Clinic had higher MCS scores than those from Charité [β = 3.91 (1.39), (1.18–6.63), p = 0.005]. Results of the independent-samples t-tests and simple regression analyses are presented in Supplementary Tables 3–5.

### Donors in caregiving roles

In 25.4% of cases, donors assumed a caregiving role post-donation, while 68% did not. Neither adjusted or unadjusted analysis found that being a caregiver donor was related to donors’ PCS. When adjusting for covariates the relationship between caregiving role and PCS remained insignificant, though center [β = 5.53 (1.42), (2.74, 8.32), p < 0.001], donor’s age [β = −0.19 (0.07), (−0.32,-0.06), p = 0.004] and time since donation [β = −0.06 (0.02), (−0.09, −0.03), p < 0.001] as covariates were found to be significantly related to PCS. Those donating at Hospital Clinic reported higher PCS than those donating at Charité, whereas older donors and donors who had undergone kidney donation longer ago presented lower PCS scores.

The donor caregiving role for the recipient was linked to lower MCS. Notably, after adjusting for age, sex, center and time since donation, the model explained 8.4% of variance of MCS compared to 1.5% found in an exploratory unadjusted regression.

Even after adjusting for covariates, the burden of caregiving was significantly associated with anxiety rates accounting for 14% of the variance. Alongside, center as a covariate demonstrated a significant association with anxiety, with those donated at Hospital Clinic reporting lower anxiety than those at Charité [β = −1.50 (0.68), (−2.84, −0.16), p = 0.03].

The burden of caregiving was significantly and positively associated with somatization rates (Table 2). After controlling for confounders, the burden of being a caregiver donor was still related to somatization rate accounted for 16.6% of variance. Among covariates sex [β = 2.07 (0.85), (0.39, 3.76), p = 0.016] and months since donation [β = 0.02 (0.01), (0.001, 0.40), p = 0.041] with female donors and longer time since donation being associated with higher somatization rate.

### Responsibility about recipient’s health

Only 41.6% of donors replied to the question regarding feelings of responsibility for the recipient’s death or health deterioration, with the question having the option of leaving it unchecked if not relevant. In total 3.2% of donors expressed feelings of responsibility, whereas 38.4% did not. Both unadjusted and adjusted analysis confirmed a significant association between feelings of responsibility and depression rates (Table 2). Importantly, the association between feelings of responsibility and depression remained significant even when center [*β* = −1.17 (0.55), (−2.25, −0.09), *p* = 0.033] and age [*β =* −0.06 (0.02), (−0.10, −0.02), *p =* 0.006] as confounders found to be significantly linked to depression.

## Discussion

The objective of the present study was to examine the long-term quality of life and psychological outcome in living kidney donors, focusing on the impact of donor-recipient relationship type, carrying a caregiver role and burden, and feeling responsible for the recipient’s health. After adjustment for gender, age, center and time since donation, the type of donor-recipient relationship was not a significant predictor of donor’s quality of life and mental health long-term. While the literature identifies parent-to-child donors experiencing significantly lower physical quality of life compared to sibling donors and child-to-parent or other genetically related donors [24, 28, 48], our study did not confirm this. Our results show no difference between groups even after pairwise comparison for physical health (PCS). However, they suggest that spouses showed lower MCS scores compared to child-to-parent donation carrying a unique psychological burden. The protective role of child-to-parent donation [31] is being moderately supported by our study, while the assumption of parenthood as a risk factor for lower QoL has not been confirmed [23, 24, 49]. Our finding regarding spouses may reflect that, contrary to their initial expectations, spousal donors often continue caregiving for their recipient post-transplant [20, 33], which maybe less of a physical strain and more of a psychological strain as they lose an equal partner and assume an unanticipated caregiving role, while also sustaining greater financial burdens and productivity losses compared to other types of donors during mutual recovery [50].

Our study found donor-caregivers scored significantly lower on the MCS with a strong and robust effect even after adjustment, whereas the PCS remained surprisingly unaffected. We assume it is less the donor-recipient-relationship type and more the burden of the caregiving role that relates to lower mental wellbeing, higher anxiety and somatization, consistent with previous linkes between caregiving responsibilities and greater long-term fatigue [10]. Caregiver donors rarely sustain the patient role for a longer period and tend to quickly return to daily responsibilities to minimize disruption, imposing additional strain not only during recovery [18, 20, 23], but also long term. While the physical wellbeing appears to recover or physical strain is not being expressed anymore as the patient role fades away, emotional strain remains as low mental wellbeing, anxiety or somatization. A bold interpretation of the result that the caregiver role predicts mental burden and that in pairwise comparison only spouses compared to child-to-parent show decreased mental wellbeing, could lie in the fact that the caregiver role is more syntonic or congruent with the parental role, whereas its conflict with spousal expectations may generate greater discomfort and disappointment.

Our study showed that assuming responsibility for the recipient’s health is linked to higher depression rates, even after adjustment. Prior studies have identified health-related concerns about the recipient as significant stressors for donors [51, 52]. Kowal et al. [53] proposed that, regardless of the nature of the donor-recipient relationship, the donors may extend their bodily self to encompass the donated kidney, enhancing perceived responsibility for its functionality, a desire to influence the recipient’s lifestyle to protect the graft, and feelings of guilt if the transplant fails [23, 43, 53]. Psychologically linking the donor’s wellbeing to the recipient’s health [23]. Our finding expand on this literature [54], suggesting that beyond the caregiving role a degree of enmeshment in the donor-recipient relationship may persist, whereby a sense of “ownership” or responsibility over the donated organ and recipient’s health translates into neurotic guilt-driven processing. This points to the emotional interpretation of these events and the degree of psychological separation from one’s donated kidney as potential determinants of donor mental health.

Our findings also pinpoint some gender differences showing that female donors demonstrate higher levels of somatization and lower levels of mental wellbeing (MCS) compared to male donors. Given that women show higher somatization baseline rates in general with a variety of psychosocial and other factors being accountable for this [55], comprise 60% of the donor population globally [56] and at least 2/3 of spousal donors [57] with an assumed high caregiver burden this difference could be explained. Also while older age and time since donation seem to be linked to lower physical wellbeing, this can be mostly attributed to natural ageing processes, as there is not other links identified.

Last, our findings suggest some differences between transplant centers with donors from the Hospital Clinic showing higher PCS and MCS scores and lower anxiety than donors from the Charité when compared for donor-recipient relationship type and caregiving role. Even though there is no specific explanation or hypothesis for this that can be based on our study design and data we assume that different centers indicate different cultural and organisational ecosystems and point to different care cultures, forms of expressing discomfort, attributing meaning and should ideally be compared to the general population of the respective countries. While we cannot explain these differences we consider them important and interesting to explore in further studies using mixed or qualitative methods.

In summary, caregiving burden and feeling responsible for the recipient’s health are the most consistent risk factors for poor mental health outcomes (anxiety, depression, somatization and MCS). Relationship type alone does not predict independently health outcomes, even though there are strong hints that spouse donors appear more vulnerable. The results highlight that even though the majority of donors appear to have a positive psychosocial and physical outcome, there remains a smaller percentage affected by the process. Thus, we consider it important for donor selection and follow-up to emphasize on assessing specific concerns, the perceived nature of the donor-recipient relationship, perceived threats and expectations related to the recipient’s condition and to donation, rather than focusing solely on the recipient’s health outcomes. Further research, eventually through a qualitative or a mixed methods lens, is needed to explore these dynamics in greater depth across donor populations.

### Limitations and implications for future research

This study has several limitations. Its cross-sectional design increases recall bias risk, and the absence of pre-donation data limits our ability to attribute outcome variations specifically to kidney donation. The small number of unrelated donors (n = 9) restricts generalizability for this subgroup. Sample selection bias is another concern, as our study is lacking data on non-responders, and donor with adverse outcomes may be less likely to participate in follow-up [58], likely overrepresenting donors with more favourable outcomes. Finally, underrepresentation of donors from culturally diverse donors due to language barriers limits insights into culturally specific experiences and outcomes. Future studies should pursue more inclusive sampling, accounting for cultural diversity and non-responder profiles.

Another key issue is the lack of standardized psychosocial assessment tools in living donation research. Despite clinical recommendations [59], no consensus or robust evidence on such tools exists, resulting in wide variability in screening practices [60]. In our study, clinical variables were assessed using internationally accepted psychometric instruments. However, their accuracy and adequacy proved ambivalent, as our independent variables failed to consistently predict mental wellbeing. The selection or development of appropriate tools standardized in donation specific outcome measures is therefore essential for future research, a finding consistent with Massey et al. [61].

The findings emphasize the importance of long-term psychosocial follow-up and support for living kidney donors, especially for a specific minority that seems to be affected. Special attention should be given to high-risk groups carrying caregiving roles and associated burden. Additionally, donor concerns and feelings of responsibility related to the recipient’s health should be carefully assessed, and addressed through appropriate counselling interventions. Enhancing donor understanding and providing targeted support are critical to safeguarding donor wellbeing.

## Data Availability

The raw data supporting the conclusions of this article will be made available by the authors, without undue reservation.
