# Supplementary material for: Beyond the gift: unveiling the impact of donor-recipient relationship on donors’ quality of life and mental health – results of a european bi-center study
Source: Transpl Int. 2026 Sep 2;39:16536. doi: 10.3389/ti.2026.16536 (PMC13580331; doi:10.3389/ti.2026.16536)
Supplement: Supplementary file 1 [file Table3.docx]

**Table 3**

Results of Student’s *t*-Test Comparing Quality of Life and Mental Health Outcomes Between Caregiver Donors and Non-Caregiver Donors (*N* = 713)

| Variable | Caregiver Donors | | Non-caregiver donors | | t | df | Cohen’s d |
| --- | --- | --- | --- | --- | --- | --- | --- |
|  | M | SD | M | SD |  |  |  |
| PCS | 82.54 | 18.15 | 84.46 | 16.72 | 1.19 | 575 | 0.11 |
| MCS | 73.91 | 20.77 | 78.95 | 16.87 | 3.19*** | 658 | 0.28 |
| PF | 86.29 | 18.21 | 88.57 | 16.75 | 1.53 | 661 | 0.13 |
| GH | 73.2 | 19.87 | 74.61 | 17.54 | 0.89 | 655 | 0.08 |
| BP | 78.58 | 25.06 | 82.76 | 22.53 | 2.05* | 655 | 0.18 |
| RP | 82.44 | 30.88 | 86.14 | 29.29 | 1.42 | 660 | 0.12 |
| SF | 85.5 | 20.63 | 90.43 | 17.86 | 3.03** | 657 | 0.26 |
| RE | 80.26 | 36.48 | 88.49 | 28.47 | 3.05** | 655 | 0.27 |
| VT | 64.07 | 20.99 | 66.95 | 19.31 | 1.66 | 660 | 0.15 |
| MH | 73.88 | 19.48 | 78.16 | 16.19 | 2.86** | 660 | 0.25 |
| PHQ-A | 2.63 | 3.63 | 1.66 | 2.91 | 3.47*** | 642 | 0.31 |
| PHQ-D | 4.23 | 4.45 | 3.24 | 3.63 | 2.93** | 662 | 0.26 |
| PHQ-S | 4.57 | 4.37 | 3.62 | 3.27 | 2.98** | 651 | 0.26 |

* *p* < .05, ** *p* < .01, *** *p* < .001
