# Supplementary material for: Beyond the gift: unveiling the impact of donor-recipient relationship on donors’ quality of life and mental health – results of a european bi-center study
Source: Transpl Int. 2026 Sep 2;39:16536. doi: 10.3389/ti.2026.16536 (PMC13580331; doi:10.3389/ti.2026.16536)
Supplement: Supplementary file 2 [file Table4.docx]

| **Table 4**  *Results of the Simple Regression Analyses Predicting Quality of Life and Mental Health from the Burden of Caregiving to the Donor (N = 713)* | | | | |
| --- | --- | --- | --- | --- |
| Burden of Caregiving to the Donor | | | | |
| Dependent Variables | *β* | *t* | *R^2^* | *F* |
| PCS | -0.45 | -0.91 | .007 | 0.83 |
| MCS | **-1.38**** | **-2.64** | **.05** | **6.95**** |
| PF | -0.27 | -0.62 | .003 | 0.39 |
| GH | **-1.20*** | **-2.44** | **.04** | **5.96*** |
| BP | **-1.64**** | **-2.68** | **.05** | **7.18**** |
| RP | -0.21 | -0.25 | .001 | 0.06 |
| SF | **-1.31*** | **-2.54** | **.05** | **6.46*** |
| VT | -0.92 | -1.71 | .002 | 2.94 |
| RE | -1.18 | -1.28 | .01 | 1.63 |
| MH | **-1.80***** | **-3.79** | **.10** | **14.33***** |
| PHQ-A | **0.24*** | **2.59** | **.052** | **6.73*** |
| PHQ-D | **0.44***** | **3.95** | **.11** | **15.60***** |
| PHQ-S | **0.24*** | **2.14** | **.03** | **4.58*** |
| ** p < ,05, ** p < ,01, *** p < ,001* | | | | |
