# Supplementary material for: Beyond the gift: unveiling the impact of donor-recipient relationship on donors’ quality of life and mental health – results of a european bi-center study
Source: Transpl Int. 2026 Sep 2;39:16536. doi: 10.3389/ti.2026.16536 (PMC13580331; doi:10.3389/ti.2026.16536)
Supplement: Supplementary file 3 [file Table5.docx]

| **Table 5**  *Results of Student’s t-Test Comparing Donors Feeling Responsible and Donors Feeling Irresponsible for Recipient’s ill Condition or Death on Quality of Life and Mental Health (N = 713).* | | | | | | | |
| --- | --- | --- | --- | --- | --- | --- | --- |
| Variable | Responsible | | Irresponsible | | *t* | *df* | Cohen’s *d* |
|  | *M* | *SD* | *M* | *SD* |  |  |  |
| PCS | 68.49 | 21.83 | 85.69 | 16.12 | **4.56***** | 269 | 1.04 |
| MCS | 67.18 | 21.04 | 78.77 | 18.07 | **2.91**** | 294 | 0.63 |
| PF | 78.91 | 22.91 | 89.55 | 16.35 | **2.89**** | 293 | 0.63 |
| GH | 61.30 | 20.90 | 76.90 | 17.26 | **4.09***** | 293 | 0.89 |
| BP | 64.89 | 30.75 | 83.65 | 21.22 | **3.91***** | 294 | 0.85 |
| RP | 65.22 | 40.41 | 87.09 | 28 | **3.46***** | 294 | 0.75 |
| SF | 80.44 | 21.92 | 89.49 | 19.04 | **2.16*** | 291 | 0.47 |
| RE | 71.01 | 41.81 | 87.95 | 29.30 | **2.56*** | 292 | 0.56 |
| VT | 56.81 | 23.38 | 67.92 | 20.17 | **2.50*** | 293 | 0.54 |
| MH | 67.78 | 17.19 | 77.92 | 17.08 | **2.73**** | 294 | 0.59 |
| PHQ-A | 3.39 | 4.46 | 1.92 | 3.07 | **2.04*** | 280 | 0.46 |
| PHQ-D | 6.01 | 5.59 | 3.33 | 3.82 | **3.10**** | 295 | 0.67 |
| PHQ-S | 6.39 | 3.99 | 3.53 | 3.69 | **3.54***** | 290 | 0.77 |
| * *p* < .05, ** *p* < .01, *** *p* < .001 | | | | | | | |
